# Supplementary material for: Grandparental partnership status and its effects on caring for grandchildren in Europe
Source: PLoS One. 2021 Mar 22;16(3):e0248915. doi: 10.1371/journal.pone.0248915 (PMC7984645; doi:10.1371/journal.pone.0248915)
Supplement: S1 File — (DOCX) [file pone.0248915.s001.docx]

**Grandparental partnership status and its effects on caring for grandchildren in Europe - Supporting Information**

The following material consists of analyses supplementing those in the paper "Grandparental partnership status and its effects on caring for grandchildren in Europe" (abbreviated in text below as *GPS&E*).

*Grandparent sex and partnership status differences in demographic attributes.*

Table 1 of *GPS&E*, duplicated below for ease of reference, presents descriptive statistics for the eight [partnership status] x [sex of grandparent] groups that are distinguished and contrasted in the paper's analyses.

**Table 1. Descriptive statistics (Duplicated from *GPS&E* Table 1).**

|  | n | Percent provided any care | Age  mean ± SD | Proximity  (8-pt scale) mean ± SD | Health  (5-pt scale) mean ± SD |
| --- | --- | --- | --- | --- | --- |
| Grandmothers |  |  |  |  |  |
| with grandfather | 18945 | 58.2 | 62.6 ± 7.2 | 4.29 ± 1.7 | 2.91 ± 1.1 |
| divorced, alone | 2662 | 56.5 | 61.4 ± 7.0 | 4.03 ± 1.7 | 2.80 ± 1.1 |
| widowed, alone | 5357 | 46.4 | 68.4 ± 8.1 | 4.42 ± 1.8 | 2.67 ± 1.1 |
| new partner | 1769 | 52.7 | 59.5 ± 6.1 | 3.72 ± 1.6 | 2.93 ± 1.1 |
| Grandfathers |  |  |  |  |  |
| with grandmother | 18091 | 49.8 | 65.6 ± 7.7 | 4.26 ± 1.7 | 2.93 ± 1.1 |
| divorced, alone | 1095 | 24.0 | 62.5 ± 7.2 | 3.77 ± 1.6 | 2.97 ± 1.2 |
| widowed, alone | 1141 | 32.3 | 70.9 ± 8.2 | 4.37 ± 1.8 | 2.75 ± 1.1 |
| new partner | 1366 | 27.8 | 63.2 ± 7.3 | 3.39 ± 1.5 | 3.07 ± 1.1 |

|  | Percent  working full time | Financial ease  (4-pt scale)  mean ± SD | Household  net worth  (1000s of Euros) median / mean | Adjusted  household income (1000s of Euros) mean ± SD |
| --- | --- | --- | --- | --- |
| Grandmothers |  |  |  |  |
| with grandfather | 23.4 | 2.77 ± 0.98 | 147.0 / 258.1 | 26.1 ± 0.28 |
| divorced, alone | 33.1 | 2.26 ± 1.01 | 28.0 / 100.8 | 15.6 ± 0.40 |
| widowed, alone | 9.9 | 2.44 ± 0.99 | 56.1 / 147.7 | 14.8 ± 0.33 |
| new partner | 39.0 | 2.88 ± 0.86 | 101.0 / 239.1 | 30.1 ± 1.03 |
| Grandfathers |  |  |  |  |
| with grandmother | 23.5 | 2.79 ± 0.99 | 150.2 / 278.9 | 28.1 ± 0.36 |
| divorced, alone | 31.1 | 2.65 ± 1.06 | 51.1 / 155.7 | 20.9 ± 0.97 |
| widowed, alone | 9.9 | 2.82 ± 1.03 | 100.1 / 235.1 | 20.8 ± 0.85 |
| new partner | 38.1 | 3.00 ± 0.95 | 138.1 / 294.7 | 35.9 ± 1.25 |

A 4 x 2 analysis of variance was conducted for each of the attributes in Table 1. The results are presented in Table S1. Every variable except for employment status (which varied in relation to partnership status, but not between the sexes) exhibited a highly significant sex difference, a highly significant partnership status difference, and a highly significant interaction between the two.

**S1 Table 1. ANOVA results for descriptive statistics.**

|  | sex of grandparent  (df = 1) | | partnership status  (df =4) | | sex x partnership status  (df = 1,4) | |
| --- | --- | --- | --- | --- | --- | --- |
|  | F | p | F | p | F | p |
| Provided care (yes/no) | 697.5 | .0000 | 211.9 | .0000 | 77.9 | .0000 |
| Age | 514.1 | .0000 | 923.1 | .0000 | 19.0 | .0000 |
| Proximity | 40.2 | .0000 | 220.4 | .0000 | 10.9 | .0000 |
| Health | 39.8 | .0000 | 51.9 | .0000 | 7.4 | .0001 |
| Employed full-time (yes/no) | 1.2 | .2707 | 308.4 | .0000 | 0.8 | .5247 |
| Financial ease | 230.2 | .0000 | 155.3 | .0000 | 65.3 | .0000 |
| Household net worth | 44.5 | .0000 | 84.0 | .0000 | 5.7 | .0007 |
| Adjusted household income | 58.7 | .0000 | 128.0 | .0000 | 5.32 | .0012 |
