# Supplementary material for: Grandparental partnership status and its effects on caring for grandchildren in Europe
Source: PLoS One. 2021 Mar 22;16(3):e0248915. doi: 10.1371/journal.pone.0248915 (PMC7984645; doi:10.1371/journal.pone.0248915)
Supplement: Attachment — Submitted filename: response to reviewers.docx [file pone.0248915.s002.docx]

Dear Dr. Helle.

Thank you for your careful critique of PONE-D-20-38604, and for the invitation to revise and resubmit it. We have revised the manuscript extensively (making the "Track Changes" version very hard to follow!), so we will briefly describe the main revisions before we provide a point-by-point response to your comments and those of the reviewers.

You criticized our analytic approach on several grounds, and we have accepted virtually all your points: (1) we have entered each of the separate measures of respondents' economic circumstances into our models rather than using a scale compounded from them; (2) we have added country dummies to all models, and (3) we have moved the analyses of the predictors of a 5-level dependent variable ("level of care") into the main manuscript, and have analyzed its predictors by means of ordered logistic regression rather than ordinary least squares regression. We should note, however, that we have also retained the logistic regression analyses involving the binary outcome variable ("ever care") and have more clearly explained our rationale: this is the outcome variable that was used in prior literature who’s results we are refuting. One consequence of these changes in the analytic structure is that all tables and the figure had to be replaced. We are pleased to report that all results remain substantively unchanged.

We have also thoroughly reorganized the introduction, in order to make the paper's aims clear from the outset and eliminate some confusions that you and one reviewer expressed.

The original submission included a Supporting Information document with three sections. One section was a presentation of the "Level of care" results, which, on your advice, have been moved to the main manuscript. Another consisted of a comparison of the performance of the compound financial status measure with its components, but since that compound measure is no longer used, this section is similarly unnecessary. The only remaining section of the original Supporting Information document is a presentation of ANOVA results documenting group differences in various descriptive statistics, which seems hardly necessary. Thus, the revised manuscript no longer has a supplementary Supporting Information document.

Finally, a couple of formatting issues may warrant comment here. Each table is presently "stacked" in two parts, simply because we were unable otherwise to fit them on the page, but Tables 2 and 3 ought ideally to be rearranged so that they are of full page width and do not have to repeat the group label as they do now, and perhaps something similar can be managed with Table 1, too. As for the Figure, it may look unchanged, but it has been redrawn because of the modified sets of predictors in the regression analyses that produced it. Also, we have produced two versions, one with error bars representing Standard Errors of the Odds Ratio estimates, and the other with 95% Confidence Intervals instead. We have no strong preference.

Below, you will find our responses to the reviews, which we have incorporated into your decision letter in a different font (Times New Roman) and colour (red).

sincerely,

Gretchen Perry & Martin Daly

From: **PLOS ONE** <[em@editorialmanager.com](mailto:em@editorialmanager.com)>
Date: Thu, Jan 21, 2021 at 2:46 AM
Subject: PLOS ONE Decision: Revision required [PONE-D-20-38406] - [EMID:99a3f79969ec2086]
To: Gretchen Cara Perry <[gretchenperry@gmail.com](mailto:gretchenperry@gmail.com)>

PONE-D-20-38406
Grandparental partnership status and its effects on caring for grandchildren in Europe.

Dear Dr. Perry,

Thank you for submitting your manuscript to PLOS ONE. After careful consideration, we feel that it has merit but does not fully meet PLOS ONE’s publication criteria as it currently stands. Therefore, we invite you to submit a revised version of the manuscript that addresses the points raised during the review process.

The reviewer #1 had some comments and pointed out several methodological concerns, which I share. In addition to those (some might overlap), I had several comments that need to be either addressed or clarified in the revised text.

1. All the hypotheses proposed in the introduction are informative/directional hypotheses but statistical testing of these hypotheses are traditional two-sided tests. The authors may want to explore the possibility that the conclusions might change given the more exact tests (reduced type II error rate).

We have made the suggested change to one-tailed tests here for a combination of reasons. First, the results supporting the hypotheses are almost all highly significant (p < .001), so little would be added by going for a more "powerful" test. Secondly, although we do indeed make directional predictions, but we are overturning contrary results in the literature, and even show that adding certain controls changes a significant negative effect of divorce on grandmaternal care into a significant positive effect, so it seems reasonable to consider, *a priori*, the possibility of effects in either direction.

Moreover, do your analyses actually test for the hypotheses #3 and #4 as these are about grandparents but your models (in Table 2) provide results by grandparent sex?

We don't understand why you ask this. In the case of hypothesis 3, the predicted contrast is highly significant in grandparents of both sexes, considered separately, so of course that shows it to be true in "grandparents". And as regards hypothesis 4, which was upheld in one sex but not in the other, what would be added by asking whether it is upheld in a pooled sample that ignores sex?

I mean you do not seem to provide any formal tests (seeing 95% CIs for odds ratios would help) for the differences of the coefficients between grandmothers and grandfathers, nor you model any interactions to examine such questions (except hypothesis #3a).

We have added 95% CIs, and hope that helps. We also hope that the revised introduction has clarified our rationale for analyzing the sexes separately. The general point is that there is reason to anticipate that marital / partnership status, and perhaps many other variables, too, will affect men and women differently in this domain. It seems to us that the most illuminating way to proceed is therefore to run separate models for men and for women and to see which predictors have similar effects on the two sexes and which do not, by direct inspection of the model results. If you were to pool all grandparents instead, and then include sex and a bunch of sex x X interaction terms as predictors, the results would surely not be as clear.

2. It was my impression that sib set size was not accounted for in any way? I understand that multilevel modeling is probably an overkill here but could a larger sib set mean increased odds that someone in the sib set was cared for? So would it make any sense to use sib set size as a predictor?

We hadn't done this, so in response to your comment, we added that variable. It turned out that it was not a significant predictor, and including it in the model neither improved fit statistics nor had any effect on any other Odds Ratio or coefficient before the fourth decimal place. So we've left it out.

3. I was unclear of the rationale to restrict the main analyses for binary response only, potentially hiding important variation in grandparental care? Furthermore, regarding to S1 Table 1 OLS regression may not be well suited here since the scale of the response is ordinal. Thus, ordered logistic (multinominal) model would probably have been more appropriate. Thus, I am not fully convinced that treating care as binary variable does not miss anything important here.

Thanks, point taken. We have switched that analysis from an OLS to an ordered logistic regression, as you suggest, and we have moved it into the main text. There is, however, a rationale for testing the binary ("Ever care") variable as well: that was the outcome measure used by Žilinčíková & Kreidl in the paper whose contrary results inspired our own analyses, and it seems important to be able to say that the turn-around in the effect of divorce on grandmothers with additional controls is not simply due to a different measure.

4. Measurement of financial status. It is unclear to me why the two measures of financial status were converted to quintiles? Was this because they were skewed? If so, why is this problematic? Regression analyses make no distributional assumptions about the independent variables. Moreover, why you think multicollinearity (which doesn’t seem too dire in this case, and might be better evaluated using variance inflation factors) would be a problem here? You are using these variables as confounders so you make no inference on them. So why bother if the SEs of these control variables are a bit off? In my opinion, just putting all three into the model is thus a better idea than using a sum score since its reliability is not too great (one probably should use coefficient omega instead of alpha, and such cut-off values are likely context-specific, see e.g. McNeish 2018) - app. 30% of its variance is measurement error, and thus you are estimating your confounder effect with error. If financial status is a true causal confounder here, you thus fail to properly account for its effect by using a proxy variable. If you choose to use your current approach, the appropriate method is to use e.g. structural equation model where you can model such measurement error in this scale.

McNeish, D. 2018 Thanks coefficient alpha, we'll take it from here. Psych. Methods 23, 412-433.

Okay, we accept your critique, and have thus abandoned the compound measure and used all four of the financial / economic circumstance variables as predictors in the revised models. However, we have retained the transformation of wealth and income to wave-specific quintiles, which we initially did not just because of skew, but because the data were collected over a 14-year period with the financial crisis in the middle, and the significance of a certain income or fortune, in euros, probably varied over waves.

5. As commented by the reviewer #1, what’s the rationale for fitting three models e.g. in Table 2? In general, you are testing a priori defined hypotheses with confounding factors and thus there is no real need for fitting multiple models or model selection. If the goal was to show the difference in results when adding more confounders, that’s fine but then why model 1?

Point taken. We have eliminated the former "model 1", but as you supposed, we indeed wanted to highlight the contrast between model 2 and 3 results: in the former, divorce appears to have a significant negative effect on grandmaternal care, but with the addition of the economic control variables, this completely flips around to become a significant positive effect

Some minor comments:

1. Is Stata's "robust cluster" approach a design-based method (like GEE)? I don’t understand line 273-275. Do you mean you computed so called “marginal effects”? If yes, what interactions got to do with these? Please clarify.

Stata's "margins" command is the one used after a logistic regression with an interaction term to generate predicted probabilities (see https://stats.idre.ucla.edu/stata/dae/using-margins-for-predicted-probabilities/). You clearly don't like the inclusion of stats-package-specific jargon, so we have dropped this sentence, but some readers sometimes like to be told what commands were used to generate what.

2. Also, please use conventional statistical terminology throughout instead of software-specific nomenclature and provide exact p-values as well as errors for the point estimates, which all are good statistical practice.

Okay, we have added exact p-values. (We agree that that's good practice, but you would be surprised how many reviewers object to "p = .000".)

3. Country should be a natural choice as a random factor in these kinds of analyses. Why you choose not to include it into the model?

Done. We have added dummy variables for country. (We do not actually show them in the results tables, however, since they would add 19 lines of clutter, and an Odds Ratio for each country relative to one arbitrarily chosen reference country would convey little of relevance to the manuscript.)

Please submit your revised manuscript by Mar 06 2021 11:59PM. If you will need more time than this to complete your revisions, please reply to this message or contact the journal office at [plosone@plos.org](mailto:plosone@plos.org). When you're ready to submit your revision, log on to <https://www.editorialmanager.com/pone/> and select the 'Submissions Needing Revision' folder to locate your manuscript file.

Please include the following items when submitting your revised manuscript:

- A rebuttal letter that responds to each point raised by the academic editor and reviewer(s). You should upload this letter as a separate file labeled 'Response to Reviewers'.
- A marked-up copy of your manuscript that highlights changes made to the original version. You should upload this as a separate file labeled 'Revised Manuscript with Track Changes'.
- An unmarked version of your revised paper without tracked changes. You should upload this as a separate file labeled 'Manuscript'.

If you would like to make changes to your financial disclosure, please include your updated statement in your cover letter. Guidelines for resubmitting your figure files are available below the reviewer comments at the end of this letter.

If applicable, we recommend that you deposit your laboratory protocols in [protocols.io](http://protocols.io/) to enhance the reproducibility of your results. Protocols.io assigns your protocol its own identifier (DOI) so that it can be cited independently in the future. For instructions see: <http://journals.plos.org/plosone/s/submission-guidelines#loc-laboratory-protocols>

We look forward to receiving your revised manuscript.

Kind regards,

Samuli Helle
Academic Editor
PLOS ONE

Journal Requirements:

When submitting your revision, we need you to address these additional requirements.

1. Please ensure that your manuscript meets PLOS ONE's style requirements, including those for file naming. The PLOS ONE style templates can be found at

<https://journals.plos.org/plosone/s/file?id=wjVg/PLOSOne_formatting_sample_main_body.pdf> and

<https://journals.plos.org/plosone/s/file?id=ba62/PLOSOne_formatting_sample_title_authors_affiliations.pdf>

2. Please provide additional details regarding participant consent. In the ethics statement in the Methods and online submission information, please ensure that you have specified (1) whether consent was informed and (2) what type you obtained (for instance, written or verbal, and if verbal, how it was documented and witnessed). If your study included minors, state whether you obtained consent from parents or guardians. If the need for consent was waived by the ethics committee, please include this information.

If you are reporting a retrospective study of medical records or archived samples, please ensure that you have discussed whether all data were fully anonymized before you accessed them and/or whether the IRB or ethics committee waived the requirement for informed consent. If patients provided informed written consent to have data from their medical records used in research, please include this information.
Once you have amended this/these statement(s) in the Methods section of the manuscript, please add the same text to the “Ethics Statement” field of the submission form (via “Edit Submission”).

For additional information about PLOS ONE ethical requirements for human subjects research, please refer to <http://journals.plos.org/plosone/s/submission-guidelines#loc-human-subjects-research>.

The data analyzed in this paper were downloaded from an anonymized, public-access survey (SHARE) from which dozens of scientific papers have been produced. We're unsure what, if anything, needs to be amended.

[Note: HTML markup is below. Please do not edit.]

Reviewers' comments:

Reviewer's Responses to Questions

**Comments to the Author**

1. Is the manuscript technically sound, and do the data support the conclusions?

The manuscript must describe a technically sound piece of scientific research with data that supports the conclusions. Experiments must have been conducted rigorously, with appropriate controls, replication, and sample sizes. The conclusions must be drawn appropriately based on the data presented.

Reviewer #1: Partly

Reviewer #2: Yes

2. Has the statistical analysis been performed appropriately and rigorously?

Reviewer #1: I Don't Know

Reviewer #2: Yes

3. Have the authors made all data underlying the findings in their manuscript fully available?

The [PLOS Data policy](http://www.plosone.org/static/policies.action#sharing) requires authors to make all data underlying the findings described in their manuscript fully available without restriction, with rare exception (please refer to the Data Availability Statement in the manuscript PDF file). The data should be provided as part of the manuscript or its supporting information, or deposited to a public repository. For example, in addition to summary statistics, the data points behind means, medians and variance measures should be available. If there are restrictions on publicly sharing data—e.g. participant privacy or use of data from a third party—those must be specified.

Reviewer #1: Yes

Reviewer #2: No

4. Is the manuscript presented in an intelligible fashion and written in standard English?

PLOS ONE does not copyedit accepted manuscripts, so the language in submitted articles must be clear, correct, and unambiguous. Any typographical or grammatical errors should be corrected at revision, so please note any specific errors here.

Reviewer #1: Yes

Reviewer #2: Yes

5. Review Comments to the Author

Please use the space provided to explain your answers to the questions above. You may also include additional comments for the author, including concerns about dual publication, research ethics, or publication ethics. (Please upload your review as an attachment if it exceeds 20,000 characters)

Reviewer #1: Interesting look at partnership status and the association with grandchild-directed caregiving. The introduction could do with tightening up – at present, it is not immediately clear what this study is addressing, nor is there a strong theoretical background. I have some concerns with the methodology, as this section is rather unclear too.

We have expanded and sharpened up our explication of the theoretical underpinnings of this research.

Comments:

From lines 38-46, it seems that grandmothering is the focus, but then the question on line 46 (regarding grandmothers) is followed with discussion on grandparents without separating the sexes (something you then criticise in the following paragraph). There are other points where grandmothering and grandparenting are used almost interchangeably (e.g. line 77-78).

We believe that the reorganization and rewriting of the introduction has solved this.

Line 39 – to help contextualise this for the reader, give examples of what would be a high-risk family situation

This request runs counter to the same reviewer's suggestion that "the introduction could do with tightening up" and we suspect that most readers will have no trouble imagining what "high-risk family situations" might be. (Addiction, incarceration, unemployment, hospitalization of the parents, etc., etc.) So we made no change here.

Line 55 – I suggest rewording the start of the paragraph as the language here might be a little strong. This previous paper (by Žilínčíkova and Kreidl) states that they attempted to include grandparental gender but “found no (statistically) significant interactions”. Their omission of these results is unusual, certainly, but I would hesitate to call their conclusions premature.

The point about wording is moot because of the way that the introduction has been restructured and rewritten. But what the reviewer says about the Žilinčíková & Kreidl paper is mistaken: they did not state that they found no significant interactions involving gender. Rather, the only interactions that they report are for [respondent marital status x country-specific divorce rate] and the only mention of gender in relation to interactions is the statement that they found no significant *three-way* interactions of [gender x respondent marital status x country-specific divorce rate]. Whether they even tried to find out if divorce might affect grandmothers and grandfathers differently is nowhere mentioned.

Line 107 – what are the theoretical grounds for expecting it in both sexes? Give brief examples here

This has now been expanded on.

Reference 29 is listed as “manuscript under review” – this should not be in the reference list as readers will not be able to find it until it has been published. One option here is to upload this paper as a citable pre-print.

The paper in question has now been accepted for publication, and the reference has been updated.

Introduction talks about uterine and agnatic grandchildren, but this is not then used in the hypotheses. Remove from here and add to methods in ‘Laterality’ section (line 214) to show why you control for laterality

Done.

These hypotheses lean heavily on the idea that divorce will result in estrangement for grandfathers – lines 90-93 “they are much more often estranged from their adult children, largely because children who were still dependent when their parents divorced are relatively likely to have been in the sole or primary custody of their mother rather than their father”. However, from the methods it does not appear that SHARE data includes information on timing of separation, so this is a dangerous a priori assumption to make.

This is not an "assumption"! It is an extensively documented fact, and we have added some references.

Similarly, “new partners” in hypotheses 3 and 4 does not appear to account for timing – one would expect that getting a new partner in the previous year vs ten years earlier may be different in the time they spend with each other versus biological grandchildren.

That is true. The duration of partnerships is an uncontrolled source of noise in the data.

Were there cases in the SHARE data included where both grandparents in a partnership were interviewed? If so, were these both included? These would be non-independent, and the current analytical structure does not appear to control for this if it has arisen as an issue.

As we stated in the original ms (and still do), both grandparents in a partnership were interviewed in Waves 1 and 2, but only one in subsequent Waves. Since we analyse the data for grandmothers and grandfathers separately, this "non-independence" issue should not be a concern.

Was any attempt made to control for the country of the respondent? The authors conclude the paper with a statement that “blanket claims...are mistaken”, yet without controlling for potential between-country differences, the claims in this paper can also be considered ‘blanket’ for Europe.

As noted earlier, we have now added country dummies to the analyses. Unfortunately, the data are insufficient to address the larger point, which is not just about country differences in rates of care, but about whether divorce and other variables have the same effects in different countries. There are several problems here, including small Ns (e.g. Ireland has fewer than ten each of divorced men and divorced women) and the confounding of country with wave (and with pre- versus post-financial crisis), and we have said what we can about this in the Discussion and Limitations sections.

Why did you divide the financial status measure into quintiles as opposed to e.g. quartiles? The financial distress measure should be simplified too for the purposes of calculation (difficult vs easy) - self-reported “with great difficulty” and “with some difficulty” (and the same for the ‘easily’ responses) is heavily subjective. The justification in the supplementary does not really justify the arbitrary way in which this whole financial status variable was created, other than by saying the results didn’t differ for grandmaternal care. What about grandfathers, since the paper is about grandparents and not just grandmothers? Is there a reason why this comparison is not presented?

The compound financial status measure has been dropped and replaced by the inclusion of all its component measures, as we noted earlier, and results for grandfathers are now complete. These two changes should deal with most of what is troubling the reviewer here, but we grant that the use of quintiles remains arbitrary; it provides an adequate degree of differentiation to show that income and wealth matter. The reviewer also asserts that the "financial ease / distress" variable "should be" reduced to a binary variable, but offers no reason for this proposal, which we reject.

It is unclear from the text what were the final model variables, and what was the justification for having three models – table 2 shows full time employment status were included, but it does not clearly mention this in the methods. On a related note, were correlations between variables checked? In particular, full-time employment and financial status may be correlated, or financial status and age.

As we noted above, we have eliminated the former model 1 and hope we have now clearly explained why we retain two models. As for correlations among predictor variables, there are indeed many, but the model results show that they are not redundant.

Odds ratios should be presented with confidence intervals to allow for interpretation

Done.

Why divide AIC by N? (e.g. table 2)

Stata provides three IC statistics: AIC, AIC/N, and BIC. AIC/N is the most convenient of the three for eye-balling the relative goodness-of-fit of (e.g.) the same model applied to different samples with different Ns, as we do in presenting separate results for grandmothers and grandfathers.

Were models 1, 2, 3 done separately for grandmothers and grandfathers?

Yes, and this should now be clearer.

Hypothesis 1 as listed on line 302 differs from hypothesis 1 from the introduction. Furthermore, it is not clear that the model structure is directly addressing the original hypothesis (direct comparison between grandmothers and grandfathers).

We have now made the exact wording identical. As for the adequacy of the "model structure" for assessing the hypothesis that divorce would have opposite effects on grandmothers and grandfathers, surely the demonstrations (a) that this is indeed so, and (b) that the two opposite effects are both highly significant, confirm exactly what was proposed, and an additional test of the difference between the two opposite-sign significant effects would be superfluous. Perhaps the addition of CIs will have made this clearer

Line 335 – your model structure means you should not state that Table 2 supports the hypothesis. The rest of the paragraph does provide support for this.

Same as last comment.

Line 420 – on the basis of the methods presented, your criticism here of this other work could equally apply to this paper.

We are baffled by this assertion. What we said at Line 420 was that Z&K had failed to separate the sexes and therefore overlooked the relatively small positive effect of divorce on grandmaternal care. We, by contrast, did look at the sexes separately, and therefore saw the difference that they missed.

Line 438 – did you control for multiple-testing here?

No. The only place where this issue applies was in the statement about Hungary, which we have removed. We have rewritten the sentence to make it a simple summary statement about directionality of effects and nothing more, so "control for multi-testing" is no longer relevant.

Reviewer #2: Thank you for this detailed exploration of the effect of grandparental care for grandchildren based on grandparents’ relationship status using the SHARE data. This is a detailed analysis that takes into consideration a number of variables that could potentially influence care provided by grandparents to grandchildren. The manuscript is well written and addresses the hypotheses well. There are no major issues that need revisions. The following are minor edits picked up during the review process. The areas for future research identified clearly and provides exciting pathways for other researchers to explore.

Lines 284-286 – the word “much” appears displaced in the sentence.

Revised. (Actually the sentence was correct, but the phrasing was perhaps a bit archaic.)

Line 302 – Consider rewording hypothesis 1 to be consistent with the introduction. “in intact couples” sounds awkward.

Done.

Line 310 – Consider stating “controlling for …” rather than “The addition of controls”

Done.
