# Supplementary material for: Grandparental partnership status and its effects on caring for grandchildren in Europe
Source: PLoS One. 2021 Mar 22;16(3):e0248915. doi: 10.1371/journal.pone.0248915 (PMC7984645; doi:10.1371/journal.pone.0248915)
Supplement: Attachment — Submitted filename: response to reviewers - revision 2.docx [file pone.0248915.s003.docx]

**From:**  <em.pone.0.713e01.73a6544c@editorialmanager.com> on behalf of PLOS ONE **Sent:** Wednesday, February 10, 2021 10:11 PM
**To:** Gretchen Perry <gretchen.perry@canterbury.ac.nz>
**Subject:**  PLOS ONE Decision: Revision required [PONE-D-20-38406R1]

Grandparental partnership status and its effects on caring for grandchildren in Europe.

Dear Dr. Perry,

Thank you for submitting your manuscript to PLOS ONE. After careful consideration, we feel that it has merit but does not fully meet PLOS ONE’s publication criteria as it currently stands. Therefore, we invite you to submit a revised version of the manuscript that addresses the points raised during the review process.

Thank you very much for your thorough revision. Prior to final acceptance, I would like you to consider couple of more changes (below) that might make your results more transparent and strengthen your conclusions (and thus adhering to PLOS ONE publication criteria).

Thank you for this prompt and encouraging response, and for your further critical comments, to which we respond, on a point-by-point basis, below:

1.  Result and conclusions related to Table 1. It is my understanding that the omnibus F-tests were significant but it is unclear actually what groups differed significantly from each other as you do not provide more detailed analyses group-specific differences. Table 1 does not currently provide clear information to make such interpretations (naturally, since it is giving just descriptive statistics), so I would like you to give more information of statistical differences (probably corrected for multiple testing) to backup your conclusions. After all, you state in the Results that: "The results in Table 1 reinforce the justification for introducing each of these variables as controls". Moreover, since some of the responses used in Table 1 are percentages, the readers might wonder why logistic regression was not used in these comparisons.

As you say, Table 1 is "just giving descriptive statistics", and it clearly indicates that there are some large differences among the eight [four partnership status x two sexes] groups in each of the tabulated variables. The differences in rates of caring might thus, in principle, be due to confoundings with other variables in the table, and that's why multivariate analyses controlling for those other variables are needed to assess the "effects" of partnership status (which is the focus of the paper). That's all we meant by the sentence you quote ("The results in Table 1 reinforce the justification for introducing each of these variables as controls"). We have rewritten the relevant paragraph in the paper to make this logic clearer.

So we're not convinced that adding the details that you request to the main mauscript would help communicate the important results. (Again, we mentioned them only to convey that there are some between-group differences in every one of the variables in Table 1, and that a multivariate approach incorporating all of them is thus warranted.) However, we recognize that there may be other readers who, like you, might wish to see such tests, so our solution is to put those additional details back into the "Supporting Information" (SI) which we removed from Revision 1, but are now reinstating for Revision 2

As regards your final sentence, we agree that logistic regression would be an appropriate way to test for group differences in the two variables that are presented as percentages (% providing care, and % employed full time), but that seems rather cumbersome, requiring that we include terms for the interactions of grandaprent sex with each of the three partnership status dummies, and it isn't easy to make a clear, simple presentation of the results of such analyses. Thus, we simply present results of (admittedly less appropriate) ANOVA analyses for these variables, too. Again, in our view, the group differences are sufficiently large and conspicuous that significance tests seem hardly necessary, and more meaningful estimation of the relative roles of these variables is provided by the multivariate analysis.

2. To be consistent (e.g. with Table 2), please consider providing 95% CIs for odds ratios instead of asterisks indicating p-value-based significance in Table 4.

Done. (In order to comply with this suggestion while keeping the dimensions of the table manageable, we have removed the repetition of Model 2 from Table 2.)

Also, we are embarrassed to note that when we prepared Revision 1, we neglected to update this Table in the same ways as Tables 2 (i.e. by replacing the compound "financial status" with its three component variables, and by adding country dummies). We have rectified this now.

3. My previous minor point #3. To account for country, you added country as a fixed effect into the model. Was there some reason why you choose not to treat country as a random factor? To me, that would have made more sense statistically and would have resulted in less parameters fitted (1 vs. 19).

We have not previously used mixed models in logistic regression so we had not thought of treating country as a single random factor as you suggest. We've tried it now, and the odds ratios, coefficients, and significance levels that we presented in Tables 1 and 2 scarcely changed. However, it appears that one cannot derive a pseudo R^2^ model fit statistic from a multilevel mixed model (https://surveydesign.com.au/docs/manuals/stata13/me.pdf), and since the basic results from the two approaches are not different, our preference is to stick with our original dummy variable approach.

Please submit your revised manuscript by Mar 27 2021 11:59PM. If you will need more time than this to complete your revisions, please reply to this message or contact the journal office at plosone@plos.org. When you're ready to submit your revision, log on to https://www.editorialmanager.com/pone/ and select the 'Submissions Needing Revision' folder to locate your manuscript file.

Please include the following items when submitting your revised manuscript:

- A rebuttal letter that responds to each point raised by the academic editor and reviewer(s). You should upload this letter as a separate file labeled 'Response to Reviewers'.
- A marked-up copy of your manuscript that highlights changes made to the original version. You should upload this as a separate file labeled 'Revised Manuscript with Track Changes'.
- An unmarked version of your revised paper without tracked changes. You should upload this as a separate file labeled 'Manuscript'.

If you would like to make changes to your financial disclosure, please include your updated statement in your cover letter. Guidelines for resubmitting your figure files are available below the reviewer comments at the end of this letter.

If applicable, we recommend that you deposit your laboratory protocols in protocols.io to enhance the reproducibility of your results. Protocols.io assigns your protocol its own identifier (DOI) so that it can be cited independently in the future. For instructions see: <http://journals.plos.org/plosone/s/submission-guidelines#loc-laboratory-protocols>

We look forward to receiving your revised manuscript.

Kind regards,

Samuli Helle
Academic Editor
PLOS ONE
